# Effects of Pigs’ Weaning Weight on Growth Performance and Blood Immunological, Antioxidant, and Gut Permeability Parameters in Early Nursery Period

**DOI:** 10.3390/ani15081119

**Published:** 2025-04-12

**Authors:** Chan Ho Kwon, Eva S. Safaie, Jannell A. Torres, Young Dal Jang

**Affiliations:** Department of Animal and Dairy Science, University of Georgia, Athens, GA 30602, USA

**Keywords:** antioxidant status, gut permeability, immunoglobulins, pigs, weaning weight

## Abstract

This study was conducted to investigate the effect of pigs’ weaning weight (WW) on their growth performance and blood immunological, antioxidant, and gut permeability parameters in the early nursery period. At weaning, a total of 48 pigs, weaned at 20.7 ± 0.74 d of age, were allotted to two WW categories—HWW, with a WW over 5.5 kg (average 6.79 ± 0.53 kg), and LWW, with a WW of less than 5.5 kg (average 4.43 ± 0.56 kg)—for a 14 d postweaning period. The WW did not affect the average daily gain (ADG) in d 0–7 postweaning or the plasma malondialdehyde levels over the entire period. HWW pigs had a higher body weight and ADG than LWW pigs (*p* < 0.05) in the overall period, with greater plasma immunoglobulin G (*p* < 0.05) and A (*p* = 0.06, tendency) levels at d 7 postweaning and superoxide dismutase activity at d 14 postweaning (*p* = 0.05, tendency), with positive correlations with the WW (*p* < 0.05). HWW pigs had lower plasma diamine oxidase (*p* < 0.05) and d-lactate (*p* = 0.06, tendency) levels at d 14 postweaning, with a negative correlation with the WW (*p* < 0.05). In conclusion, although there was no effect of the WW on growth rate in the first week postweaning and oxidative stress in the early nursery period, HWW pigs exhibited greater growth performance, immunoglobulin levels, and antioxidant capacity but lower gut permeability than LWW pigs in the early nursery period.

## Simple Summary

Due to increasing litter sizes in modern hyperprolific sows, the weaning weight may decrease, resulting in pigs becoming more prone to weaning stress. A study was conducted to evaluate how pigs’ weaning weight affects their growth, immunity, antioxidant, and gut permeability parameters with 48 pigs who were weaned at 21 d of age in a 14 d growth trial. High-weaning-weight pigs had a greater growth rate than low-weaning-weight pigs in the overall early nursery period, although the growth rate was similar in the first week postweaning. High-weaning-weight pigs had higher blood immunoglobulin levels at d 7 postweaning, while they had a better antioxidant status and lower gut permeability at d 14 postweaning compared with low-weaning-weight pigs. The weaning weight was positively correlated with antioxidant status and immunoglobulin levels after weaning, but negatively correlated with gut permeability. Although the weaning weight did not affect the growth rate of pigs in the first week after weaning, a high weaning weight could result in a high growth rate, antioxidant status, and immunoglobulin levels, while lowering the gut permeability in the early nursery period.

## 1. Introduction

Weaning is one of the most stressful events in a pig’s life, as it causes inflammation, oxidative stress, and gut health issues such as dysbiosis and leaky gut, evidenced by increased proinflammatory cytokines, such as tumor necrosis factor-α, interleukin-1β, and interleukin-6; oxidative stress, such as malondialdehyde and protein carbonyl levels; and reduced gene expression of tight junction proteins such as zonula occluden-1 (ZO-1), Occludin, and Claudin in pigs [1,2]. To help overcome weaning stress in pigs, various nutritional strategies have been implemented, such as reducing dietary protein levels or utilizing feed additives like probiotics, organic acids, and medium-chain fatty acids, all of which have been reported to have positive effects during the postweaning period [3].

However, due to increasing litter sizes in modern hyperprolific sows, pigs’ birth weight decreases, resulting in a low weaning weight (WW) [4]. Low-WW pigs may have a less mature digestive system, underdeveloped gut microbiota, and impaired gut health compared to heavy-WW pigs, which can result in high morbidity and mortality [5,6]. The negative impact of weaning may be more significant when pigs are weaned at an earlier age than later [5,7,8], and a higher weaning age has positive effects on growth, immunity, and antioxidant parameters, which may result from the more developed gut structure, microbiota, antioxidant system, and enzyme activities of pigs with a heavier WW [5,8,9]. However, increasing the weaning age presents several challenges, including implications for sow management, pig flow, and facility requirements [10]. As a result, increasing the WW and managing pigs based on their WW could offer more practical approaches, as these measures can improve piglets’ health and growth rates without requiring significant changes to the overall production system [10,11]. However, there is limited information regarding how the WW influences the overall systemic health of nursery pigs when they are weaned early at 21 d of age, which is the current average weaning age in the United States (average: 20.5 days of age) [12,13]. In addition, several previous studies have mainly focused on the effect of the WW on the production performance during the nursery period and/or throughout the entire lifetime [14,15,16,17]. Therefore, it is important to know how the WW is associated with pig health parameters such as immunity, antioxidant capacity, oxidative stress, and gut permeability and their relationships with the WW, which can provide crucial information for more successful nutritional strategies and management decisions for early-weaned pigs of various WWs. Therefore, this study was conducted to evaluate the effect of the WW of pigs on growth performance and blood immunological, antioxidant, and gut permeability parameters in the early nursery period, with the hypothesis that high-WW (HWW) pigs may have higher blood immunoglobulin levels and antioxidant capacity and lower gut permeability and oxidative stress than low-WW (LWW) pigs in the early postweaning period.

## 2. Materials and Methods

### 2.1. Animals, Experimental Design, Diets, and Housing

At weaning at 20.7 ± 0.74 d of age, a total of 48 pigs (Camborough × PIC337; 24 barrows and 24 gilts) from 9 sow litters were selected based on their WW and health condition. Each sow litter contributed 2–5 piglets to each WW category. All piglets were allotted to 2 WW categories in 3 replicate pens per treatment, with 8 pigs (4 barrows and 4 gilts) per pen, which were balanced by breed, sex, and littermate in a randomized complete block design for a 14 d postweaning period. The treatments were as follows: (1) HWW, with a high WW of over 5.5 kg (average initial WW: 6.79 ± 0.53 kg, 5.9 to 7.9 kg; average birth weight: 1.66 ± 0.31 kg), and (2) LWW, with a low WW of less than 5.5 kg (average initial WW: 4.43 ± 0.56 kg, 3.6 to 5.3 kg; average birth weight: 1.30 ± 0.23 kg).

Before weaning, pig processing including 200 mg of iron dextran injection (UNIFERON^®^ 200, Pharmacosmos, Inc., Watchung, NJ, USA) into the neck muscle, ear notching, and tail docking and castration was performed under the standard operational protocol of the University of Georgia swine farm by trained personnel. No creep feed was provided for pigs during the suckling period. At weaning, piglets were transferred from the farrowing facility to the nursery facility, located next to the farrowing building, using a small cart. The transfer took less than two minutes, minimizing transportation stress. All pigs were housed in nursery pens (2.0 m × 2.0 m) with plastic flooring and had free access to water and feed in an environmentally controlled nursery facility at the University of Georgia Swine Research Unit. A common corn–soybean meal-based nursery diet was formulated to meet or exceed the NRC [18] nutrient requirement estimates and was provided to all pigs for the whole experimental period (Table 1).

### 2.2. Data and Sample Collection and Blood Analysis

The pigs were individually weighed at the start of the trial and on d 7 and 14 postweaning to calculate the average daily gain (ADG), and feed disappearance was recorded when the pigs were weighed to calculate the average daily feed intake (ADFI) and gain-to-feed ratio (G:F).

For blood samples, 12 pigs per treatment were selected (4 pigs per pen, with 2 barrows and 2 gilts within each pen) based on the average body weight (BW) within each treatment and sow litter origin. Blood samples (10 mL) were collected at d 7 and 14 postweaning via jugular venipuncture in disposable vacutainer tubes containing K3 EDTA (Becton Dickinson, Franklin, NJ, USA) as an anticoagulant. Plasma samples were obtained from blood by centrifugation at 2500× *g* for 30 min at 4 °C and stored at −80 °C until analysis. Due to the high degree of hemolysis, two samples were excluded from the laboratory analysis on each sampling day. Plasma samples were analyzed for superoxide dismutase (SOD) activity and malondialdehyde (MDA) levels using colorimetric kits (Cayman Chemical Company, Ann Arbor, MI, USA), diamine oxidase (AFG Bioscience Northbrook, IL, USA), d-lactate (BioAssay Systems, Hayward, CA, USA), and immunoglobulin (Ig) G and A (Bethyl Laboratories, Montgomery, TX, USA) using ELISA kits with a spectrophotometer (Thermo Fisher Scientific, Waltham, MA, USA).

### 2.3. Statistical Analysis

All data obtained in the current study were analyzed by ANOVA, using the PROC MIXED procedure of SAS (ver. 9.4. SAS Inst. Inc., Cary, NC, USA). All data were tested for normality by the Shapiro–Wilk test. A pen was used as an experimental unit for growth performance, and the models included the WW category as a fixed effect, with a replicate as a random effect. An individual pig was used as an experimental unit for blood parameters, and the models included the WW category as a fixed effect, with a replicate within the pen and a full pen as random effects. Repeated measures ANOVA was used to evaluate the day effect and WW category × day interaction. Pearson correlation coefficients between the BW and ADG and between growth (BW and ADG) and blood parameters (plasma SOD, MDA, d-lactate, diamine oxidase, IgA, and IgG) were determined using PROC CORR of SAS with individual values. The least square means were separated using the PDIFF option in SAS. Statistical differences were established at *p* ≤ 0.05, and tendencies were established at *p* ≤ 0.10.

## 3. Results

### 3.1. Growth Performance

In the period of d 0–14 postweaning, HWW pigs always had a greater BW at d 0, 7, and 14 postweaning and a greater ADG in d 7–14 and 0–14 postweaning than LWW pigs (*p* < 0.05), while the ADG in the period of d 0–7 postweaning was not different between HWW and LWW pigs (Table 2). The WW did not affect the ADFI until d 14 postweaning (0.244 and 0.214 kg/d for HWW and LWW pigs in the period of d 0–14 postweaning, respectively). The G:F was greater in HWW pigs than in LWW pigs in the period of d 0–14 postweaning (*p* < 0.05; 0.688 and 0.596 for HWW and LWW pigs in the period of d 0–14 postweaning, respectively).

The BW at d 0 (weaning), 7, and 14 postweaning and ADG in the d 7–14 and 0–14 postweaning periods were positively correlated with each other (*p* < 0.05; Table 3). Although the ADG in the period of d 0–7 postweaning had positive correlations with the BW at d 7 and 14 postweaning and the ADG in the period of d 0–14 postweaning (*p* < 0.05), it had no correlation with the BW at d 0 postweaning and the ADG in the period of d 7–14 postweaning.

### 3.2. Plasma IgG and A Levels

On d 7 postweaning, HWW pigs had greater IgG (*p* < 0.05) and A (*p* = 0.06, tendency) levels than LWW pigs, while no differences were observed on d 14 postweaning (Table 4). Day effects were observed in plasma IgG and A levels, with the plasma IgG levels decreasing from d 7 to 14 postweaning (*p* < 0.05), while the plasma IgA levels increased during the same period (*p* < 0.05).

### 3.3. Plasma Diamine Oxidase and D-Lactate Levels

HWW pigs had lower plasma d-lactate (*p* = 0.06, tendency) and diamine oxidase (*p* < 0.05) levels than LWW pigs on d 14 postweaning (Table 5). A day effect was observed in the plasma d-lactate levels, with a significant increase from d 7 to 14 postweaning (*p* < 0.05), while no day effect was observed in the plasma diamine oxidase levels.

### 3.4. Plasma SOD and MDA Levels

At d 14 postweaning, HWW pigs tended to have greater plasma superoxide dismutase activity than LWW pigs (*p* = 0.05, tendency), with no difference in plasma malondialdehyde levels (Table 6). A day effect was observed in the plasma MDA levels, which decreased from d 7 to 14 postweaning (*p* < 0.05), while no day effect was observed in the plasma SOD activity.

### 3.5. Correlation Analysis Between Postweaning Growth and Blood Parameters

The WW (BW at d 0 postweaning) had positive correlations with the plasma SOD at d 7 (*p* = 0.09, tendency) and 14 (*p* < 0.05) postweaning and IgG and A levels at d 7 postweaning (*p* < 0.05), while it showed a negative correlation with the plasma d-lactate levels at d 14 postweaning (*p* < 0.05; Table 7).

After weaning, there were positive correlations of the plasma SOD activity at d 14 postweaning with the BW at d 7 and 14 postweaning and ADG in the periods of d 7–14 and 0–14 postweaning (*p* < 0.05; Table 7), while the plasma d-lactate levels at d 14 postweaning were negatively correlated with the BW at d 7 and 14 postweaning (*p* < 0.05). The plasma d-lactate levels at d 7 postweaning had a positive correlation with the ADG in the period of d 0–7 postweaning (*p* < 0.05). No correlation was observed between growth and plasma MDA or diamine oxidase levels.

## 4. Discussion

Weaning stress negatively impacts the postweaning growth and health of pigs, as it increases inflammation, oxidative stress, and gut dysbiosis and thereby the incidence of postweaning diarrhea [1,2]. Therefore, it is important to understand the consequences of weaning stress in pigs during the early nursery period to ensure a smooth weaning transition and apply nutritional strategies to alleviate these negative impacts, ultimately improving postweaning growth and health. Previous studies reported that increasing the weaning age may alleviate weaning stress, as it results in a more developed immune, intestinal, and antioxidant defense system with heavier WW [8,9]. However, there is limited information on how the WW affects the systemic health of pigs who are weaned early at around 3 weeks of age, as previous studies mainly focused on the effect of the WW on their life-time production performance [14,16,17]. Blood markers have been widely used in swine nutrition research, because they do not require animal sacrifice to collect tissues and digesta samples for measuring certain health-related parameters [19]. Thus, understanding the relationship between the WW and blood parameters in pigs is also important, as using these markers is more efficient for assessing the health status of pigs after weaning and can help develop targeted nutritional strategies. Therefore, the current study evaluated the effect of the WW on growth performance and blood parameters related to immunity, antioxidant status, and gut permeability, as well as their relationship with the WW.

In the overall early nursery period (d 0–14 postweaning), HWW pigs exhibited significantly greater growth rates than LWW pigs. This resulted in greater BWs in HWW pigs at d 14 postweaning than in LWW pigs, with a greater difference (2.84 kg) between HWW and LWW pigs than that at weaning (2.36 kg) in a 2-week period. This result agrees with previous studies reporting that heavier WWs led to greater growth rates in the nursery period [14,16,17]. HWW pigs may have a more developed gastrointestinal tract structure and function than LWW pigs [5,14], resulting in an increased growth rate in the early nursery period.

Interestingly, there was no significant difference in growth rate during d 0–7 postweaning. In addition, there was no correlation between the WW and the growth rate in the first week after weaning, nor between the growth rates of the first and second weeks, although the growth rate in the second week was positively correlated with the WW. This result agrees with previous studies reporting that pigs grow similarly immediately after weaning, regardless of their WW [14,15]. Ming et al. [9] also reported that increasing the weaning age did not affect the growth rate in the first week after weaning. Therefore, this result indicates that weaning stress immediately following weaning affects pigs’ growth in a similar manner across all pigs, regardless of their WW.

After the first week of weaning, HWW pigs began to show a greater growth rate than LWW pigs, and the WW had positive correlations with the growth rate in the periods of d 7–14 and 0–14 postweaning. These results agree with previous studies [9,14,15], which reported an increased growth rate with an increasing weaning age and WW after the first 1 or 2 weeks postweaning. This suggests that HWW pigs may overcome weaning stress immediately after weaning more easily than LWW pigs, likely due to their more developed physiological system, as low-birth-weight pigs may have delayed gut maturation during the suckling period compared with normal-birth-weight pigs, resulting in lower digestive capacity at weaning [20], which can cause a reduction in weight gain after weaning. Holanda et al. [6] also reported that LWW pigs had increased intestinal oxidative stress and inflammation, as well as immature microbiota, compared with HWW pigs at weaning. Therefore, these results indicate that all pigs experience weaning stress during the first week, but increasing the WW could result in a greater growth rate in the overall early nursery period, with improvements becoming evident after the first week of weaning. However, further large-scale studies are needed to demonstrate the effect of the WW on growth performance and its association not only with blood parameters, but also with intestinal and tissue markers related to overall health.

For blood parameters, HWW pigs had higher immunoglobulin G and A levels in their blood at d 7 postweaning compared to LWW pigs. HWW pigs are more likely to have a higher birth weight, resulting in a high colostrum intake at birth [21,22], which can increase plasma IgG and A levels, which remain high in the blood until d 7 postweaning, as Rooke et al. [23] reported that high plasma IgG levels in early life (d 2–7 of age) resulted in high plasma IgG levels until weaning (d 28 of age). In addition, increased colostrum intake resulted in a higher weaning weight, with a positive correlation between colostrum intake and plasma IgG levels at weaning [24]. Therefore, these results suggest that a higher birth weight and, consequently, WW are crucial for maintaining higher passive immunity immediately after weaning. Interestingly, the significant differences observed at d 7 postweaning for both plasma IgG and A levels disappeared at d 14 postweaning. This result indicates that the passive immunity from colostrum may not be very effective by d 14 postweaning, which corresponds to approximately d 35 of age in the current study. Frenyó et al. [25] reported a mean half-life of serum IgG in piglets of 9.73 days. Based on this, it can be inferred that at d 35 of age (14 d postweaning), the remaining passive immunity in the blood of weaning pigs would be approximately 10% of the original value. In addition, the plasma IgG levels decreased from d 7 to 14 postweaning, which is consistent with previous studies reporting that the blood IgG levels from colostrum, which provides passive immunity, decrease as pigs grow [26,27]. However, the plasma IgA levels increased from d 7 to 14 postweaning, which agrees with previous studies [27,28,29]. Sugiharto et al. [30] also reported an increase in plasma IgA levels after weaning with an *E. coli* F18 challenge. IgA is also produced in the intestinal mucosa in response to antigen exposure in the gut, triggered by the transition to solid feed and introduction of new feed components, which may stimulate an immune response [31], resulting in an increase in plasma IgA levels from d 7 to 14 postweaning. Vaerman et al. [32] reported that the gut lymph contributes IgA to the blood, with 73.9% originating from local synthesis in the gut, meaning that 30.8% of the intravascular IgA pool is supplied daily by intestinally synthesized IgA. Therefore, the increase in plasma levels from d 7 to 14 postweaning can be attributed to the immune response that is triggered by weaning-related challenges in the gut, which stimulate local IgA synthesis and contribute to its elevation in the bloodstream.

Blood diamine oxidase and d-lactate levels are valid markers of gut permeability, as their concentrations increase when the gut permeability is elevated [33]. In the current study, the plasma diamine oxidase and d-lactate levels were greater in LWW pigs than HWW pigs at d 14 postweaning. This result indicates that LWW pigs may be more significantly affected by weaning stress in terms of gut permeability. Although there is limited information available regarding the effect of the WW on gut permeability, Tsukahara et al. [34] reported that early weaning results in a less mature digestive system than late weaning, characterized by lower digestive enzyme activity and reduced villi height. Also, it has been reported that pigs who were weaned at d 21 of age showed higher plasma d-lactate and diamine oxidase levels at d 28 to 56 of age compared with those who were weaned at d 28, 35, and 42 of age [35]. This suggests that LWW pigs may have compromised gut barrier function, making them more susceptible to increased gut permeability, which is associated with intestinal health challenges, compared with HWW pigs. In addition, this result corresponds with the results for the postweaning tight junction protein expression, in which increasing the weaning age led to higher gene expressions of tight junction protein in the intestine [35], which also supports the use of blood diamine oxidase and d-lactate levels as reliable markers for assessing gut permeability in pigs after weaning. Although there was no significant difference in plasma diamine oxidase levels between d 7 and 14 postweaning, the plasma d-lactate levels increased from d 7 to 14 postweaning. This result agrees with previous studies suggesting that gut permeability may increase in the early nursery period [33,36], indicating that pigs may face continuous challenges in their gut during this phase.

In the current study, the plasma SOD activity and MDA levels were analyzed as key markers to investigate the oxidative status [37]. There was no significant difference in plasma MDA levels, which are a marker for lipid peroxidation, indicating oxidative stress, which agrees with Michiels et al. [20], who reported that the plasma MDA levels were not different in the d 18–28 postweaning period between low- and normal-birth-weight pigs with significantly different WWs (5.5 vs. 7.1 kg). This result suggests that pigs may have similar oxidative stress levels, regardless of their WW in the early nursery period, which could explain the lack of a difference in growth rate in the first week postweaning. However, the plasma SOD activity was greater in HWW pigs than LWW pigs at d 14 postweaning. Michels et al. [20] reported that the plasma glutathione peroxidase levels during d 18–28 postweaning were higher in normal-birth-weight pigs than in low-birth-weight pigs. This result indicates that LWW pigs may have a reduced antioxidant capacity, as indicated by the lower SOD activities, potentially due to delayed development of the oxidative defense system [20]. Although there was no day effect on the plasma SOD activity, the plasma MDA levels decreased from d 7 to 14 postweaning. This result aligns with previous findings [38] reporting that the plasma MDA levels of pigs at d 14 of age increased after weaning, with a peak at d 3 postweaning, and decreased thereafter. This result indicates that oxidative stress can be elevated immediately after weaning during the first week due to weaning stress, while pigs are likely adapting to the stressors of the nursery period, resulting in a reduction in oxidative stress by d 14 postweaning [2]. Since the current study only analyzed plasma SOD activities and MDA levels to assess oxidative status, further studies with multiple markers of oxidative status are needed to provide a more comprehensive understanding of the impact of the WW on the antioxidant capacity and oxidative stress in weaning pigs.

In the current study, for a more comprehensive understanding of the relationship between the WW and various blood parameters, a correlation analysis was performed. There was a positive correlation between the WW and plasma SOD activity at d 7 and 14 postweaning and plasma IgG and A at d 7 postweaning, with a negative correlation between the WW and plasma d-lactate level at d 14 postweaning. Although there is limited information available, this result indicates that increasing the WW can result in improved antioxidant and immune status and a reduced possibility of leaky gut in the weaning transition period, as increasing the birth weight or weaning age could enhance the antioxidant capacity, immunity, and gut maturation [20,21,35]. Interestingly, the plasma SOD activity at d 14 postweaning was positively correlated with the BW at d 0, 7, and 14 postweaning and ADG in the periods of d 7–14 and 0–14 postweaning, while the plasma d-lactate levels at d 14 postweaning showed a negative correlation with the BW at d 0, 7, and 14 postweaning. However, other blood parameters, including plasma MDA, IgG, and A levels, did not show any correlation with the BW and ADG at d 7 and 14 postweaning. These results indicate that after weaning, the antioxidant capacity and gut permeability may be more closely linked to a pig’s body size and growth rate after the first week of weaning than oxidative stress and immune parameters. However, further studies are needed to investigate how the body size and postweaning growth rate affect physiological development and to determine whether the WW or age has a greater impact on these parameters. Additionally, it is important to explore the relationship between stress levels, measured by physiological stress markers such as cortisol and lactate, and pigs’ health after weaning.

In addition, the plasma d-lactate levels at d 7 postweaning were positively correlated with the growth rate in the period of d 0–7 postweaning, although the growth rate in this period was not correlated with the WW. This result indicates that pigs with greater growth rates immediately after weaning may have greater gut permeability than pigs with lower growth rates, regardless of their WW. Previous studies reported that increased early feed consumption after weaning may increase the challenges in the gut, resulting in increased postweaning diarrhea and gut inflammation due to undigested and unabsorbed dietary components, which can result in impaired intestinal barrier functions, although they may grow faster than those not consuming feed at high level [26,33]. This result suggests that the gut permeability immediately after weaning is more likely to be positively associated with the growth rate in that period, possibly reflecting the feed intake rather than WW or growth rate in the following week.

## 5. Conclusions

In the current study, it was demonstrated that increasing pigs’ WW could enhance their growth rate, antioxidant capacity, gut integrity, and immunity in the early nursery period, despite the fact that weaning stress immediately after weaning may be similar across all pigs, regardless of their WW. Additionally, the WW was positively correlated with plasma SOD activities and immunoglobulin levels but negatively correlated with gut permeability in the early nursery period. Therefore, it is crucial to focus on increasing pigs’ birth weight and promoting weight gain during the suckling period by providing creep feed, which not only supplies nutrients but also reduces the stress of feed transition at weaning, ultimately leading to higher WWs. Further studies are needed to investigate the combined effects of the WW at different weaning ages on growth performance and health-related parameters, in order to better understand how the WW and weaning age are associated with pigs’ development and health outcomes.

## Figures and Tables

**Table 1:** Diet formulation and calculated chemical composition ^1^.

Ingredients, %	d 0–14 Postweaning
Corn	26.71
Soybean meal (48% crude protein)	14.64
Whey, dried	15.00
Oats	10.00
HP300 ^1^	10.00
Lactose	10.00
Fish meal	5.00
Animal plasma	3.00
Soybean oil	2.96
L-Lysine-HCl	0.30
DL-Methionine	0.11
L-Threonine	0.06
Dicalcium phosphate	0.47
Limestone	0.85
Salt	0.50
Trace mineral mix ^2^	0.15
Vitamin mix ^3^	0.25
Calculated chemical composition	
Metabolizable energy (kcal/kg)	3444
Crude protein (%)	23.68
SID ^4^ lysine (%)	1.51
SID methionine + cysteine (%)	0.78
Total Ca (%)	0.83
STTD ^4^ P (%)	0.42

^1^ Hamlet Protein, Findlay, OH. ^2^ The trace mineral premix supplied the following per kilogram of diet: 33 mg of Mn as manganous oxide, 110 mg of Fe as ferrous sulfate, 110 mg of Zn as zinc sulfate, 16.5 mg of Cu as copper sulfate, 0.3 mg of I as Ca iodate, and 0.3 mg of Se as sodium selenite. ^3^ The vitamin premix supplied the following per kilogram of diet: 11,000 IU of vitamin A, 2000 IU of vitamin D_3_, 99 IU of vitamin E, 4.4 mg of vitamin K, 55 µg of vitamin B_12_, 9.9 mg of riboflavin, 31.9 mg of pantothenic acid, 55 mg of niacin, 0.9 mg of folic acid, 3.9 mg of vitamin B_6_, 3.1 mg of thiamin, and 0.3 mg of biotin, and 600 mg of choline chloride. ^4^ SID = standardized ileal digestible; STTD = standardized total tract digestible.

**Table 2:** Postweaning growth performance of pigs in different weaning weight (WW) categories ^1^.

	Treatment ^2^			
Item	HWW	LWW	SEM	*p*-Value
Weaning age	20.72	20.88	0.36	0.22
BW, kg				
d 0	6.79	4.43	0.15	<0.01
d 7	7.59	5.21	0.21	<0.01
d 14	9.04	6.20	0.26	<0.01
ADG, kg/d				
d 0–7	0.114	0.111	0.013	0.88
d 7–14	0.208	0.142	0.027	0.01
d 0–14	0.161	0.126	0.010	0.04

^1^ *n* = 3 pens per treatment. ^2^ Treatments were (1) HWW, high WW of over 5.5 kg (average initial weight: 6.79 ± 0.53 kg), and (2) LWW, low WW less than 5.5 kg (average initial weight: 4.43 ± 0.56 kg).

**Table 3:** Pearson correlation coefficients between postweaning body weight (BW) and average daily gain (ADG).

Item	d 0 BW	d 7 BW	d 14 BW	d 0–7 ADG	d 7–14 ADG
d 7 BW	0.954				
d 14 BW	0.909	0.939			
d 0–7 ADG	0.158 ^1^	0.447	0.382		
d 7–14 ADG	0.383	0.365	0.663	0.059 ^1^	
d 0–14 ADG	0.396	0.537	0.743	0.586	0.844

^1^ No significant differences. Otherwise, significantly different (*p* < 0.05).

**Table 4:** Plasma immunoglobulin (Ig) G and A levels of pigs in different weaning weight (WW) categories at d 7 and 14 postweaning ^1^.

	Treatment ^2^			
Item	HWW	LWW	SEM	*p*-Value
IgG, mg/mL				
d 7 ^3^	9.44	6.07	1.030	0.05
d 14	5.14	5.02	0.646	0.86
IgA, mg/mL				
d 7 ^3^	0.164	0.138	0.008	0.06
d 14	0.230	0.222	0.017	0.65

^1^ *n* = 10 per treatment. ^2^ Treatments were (1) HWW, high WW of over 5.5 kg (average initial weight: 6.79 ± 0.53 kg), and (2) LWW, low WW of less than 5.5 kg (average initial weight: 4.43 ± 0.56 kg). ^3^ No day × WW category interaction. Day effect (*p* < 0.05) in IgG and A based on repeated measures ANOVA.

**Table 5:** Plasma d-lactate and diamine oxidase levels of pigs in different weaning weight (WW) categories at d 7 and 14 postweaning ^1^.

	Treatment ^2^			
Item	HWW	LWW	SEM	*p*-Value
D-lactate, mM				
d 7 ^3^	0.58	0.44	0.160	0.26
d 14	0.74	1.46	0.227	0.06
Diamine oxidase, ng/mL				
d 7 ^3^	36.77	34.87	4.182	0.70
d 14	27.42	37.15	4.134	0.04

^1^ *n* = 10 per treatment. ^2^ Treatments were (1) HWW, high WW of over 5.5 kg (average initial weight: 6.79 ± 0.53 kg), and (2) LWW, low WW of less than 5.5 kg (average initial weight: 4.43 ± 0.56 kg). ^3^ No day × WW category interaction. Day effect (*p* < 0.05) in d-lactate based on repeated measures ANOVA.

**Table 6:** Plasma malondialdehyde (MDA) levels and superoxide dismutase (SOD) activity of pigs in different weaning weight (WW) categories at d 7 and 14 postweaning ^1^.

	Treatment ^2^			
Item	HWW	LWW	SEM	*p*-Value
SOD, U/mL				
d 7 ^3^	4.92	4.26	0.310	0.16
d 14	5.07	3.97	0.387	0.05
MDA, µM				
d 7 ^3^	7.20	7.31	0.652	0.91
d 14	5.94	5.71	0.690	0.65

^1^ *n* = 10 per treatment. ^2^ Treatments were (1) HWW, high WW of over 5.5 kg (average initial weight: 6.79 ± 0.53 kg), and (2) LWW, low WW of less than 5.5 kg (average initial weight: 4.43 ± 0.56 kg). ^3^ No day × WW category interaction. Day effect (*p* < 0.05) in MDA based on repeated measures ANOVA.

**Table 7:** Pearson correlation coefficients between postweaning growth and blood parameters ^1^.

	SOD, U/mL		MDA, µM		D-Lac, mM		DAO, ng/mL		IgG, mg/mL		IgA, mg/mL	
Item	d 7	d 14	d 7	d 14	d 7	d 14	d 7	d 14	d 7	d 14	d 7	d 14
d 0 BW ^2^	0.394 **	0.466 *	0.042	0.111	0.207	−0.572 *	0.101	−0.360	0.420 **	0.138	0.414 **	0.112
d 7 BW	0.342	0.526 *	−0.008	0.219	0.410	−0.576 *	0.020	−0.391	0.324	0.162	0.371	0.110
d 14 BW	0.343	0.680 *	0.032	0.240	0.366	−0.518 *	0.044	−0.396	0.341	−0.016	0.230	0.161
d 0–7 ADG	−0.019	0.336	−0.117	0.314	0.753 *	−0.106	−0.181	−0.165	−0.159	0.097	0.000	0.028
d 7–14 ADG	0.170	0.629 *	0.092	0.158	0.088	−0.196	0.067	−0.139	0.166	−0.342	−0.147	0.177
d 0–14 ADG	0.127	0.717 *	0.006	0.312	0.420	−0.217	−0.051	−0.219	0.043	−0.229	−0.121	0.160

^1^ BW: body weight; ADG: average daily gain; SOD: superoxide dismutase; MDA: malondialdehyde; D-Lac: d-lactate; DAO: diamine oxidase; IgG: immunoglobulin G; IgA: immunoglobulin A. ^2^ Significant differences: * *p* < 0.05, ** *p* < 0.10.

## Data Availability

The raw data supporting the conclusions of this article will be made available by the authors on request.

## Abbreviations

The following abbreviations are used in this manuscript:

ADG	Average daily gain
ADFI	Average daily feed intake
BW	Body weight
G:F	Gain-to-feed ratio
HWW	High weaning weight
Ig	Immunoglobulin
LWW	Low weaning weight
MDA	Malondialdehyde
SOD	Superoxide dismutase
WW	Weaning weight
ZO	Zonula occluden
